# *TOX* and *ADIPOQ* Gene Polymorphisms Are Associated with Antipsychotic-Induced Weight Gain in Han Chinese

**DOI:** 10.1038/srep45203

**Published:** 2017-03-22

**Authors:** Shen Li, Chengai Xu, Yuan Tian, Xueshi Wang, Rui Jiang, Miaomiao Zhang, Lili Wang, Guifu Yang, Ying Gao, Chenyu Song, Yukun He, Ying Zhang, Jie Li, Wei-Dong Li

**Affiliations:** 1 Department of Genetics, College of Basic Medical Sciences, Tianjin Medical University, Tianjin, 300070, China; 2 Department of Psychiatry, College of Basic Medical Sciences, Tianjin Medical University, Tianjin, 300070, China; 3 Tianjin Mental Health Centre, Tianjin Anding Hospital, Tianjin, 300222, China; 4 Tianjin Jianhua Hospital, Tianjin, 300112, China

## Abstract

To find the genetic markers related to the antipsychotic-induced weight gain (AIWG), we analyzed associations among candidate gene single-nucleotide polymorphisms (SNPs) and quantitative traits of weight changes and lipid profiles in a Chinese Han population. A total of 339 schizophrenic patients, including 86 first-episode patients (FEPs), meeting the entry criteria were collected. All patients received atypical antipsychotic drug monotherapy and hospitalization and were followed for 12 weeks. Forty-three SNPs in 23 candidate genes were calculated for quantitative genetic association with AIWG, performed by PLINK. The *TOX* gene SNP rs11777927 (*P* = 0.009) and the *ADIPOQ* gene SNP rs182052 (*P* = 0.019) were associated with AIWG (in body mass index, BMI). In addition, the *BDNF* SNP rs6265 (*P* = 0.002), *BDAF* SNP rs11030104 SNP (*P* = 0.001), and *ADIPOQ* SNPs rs822396 (*P* = 0.003) were significantly associated with the change of waist-to-hip ratio (WHR) induced by atypical antipsychotics. These results were still significant after age and gender adjustments. These findings provide preliminary evidence supporting the role of *TOX, ADIPOQ* and *BDNF* in weight and WHR gain induced by atypical antipsychotics.

Schizophrenia is a complex and devastating psychiatric disorder that occurs worldwide, and for decades it was generally thought to have a uniform lifetime risk of approximately 1% across time, geography and sex^1^. Obesity and overweight affect 30–70% of patients with schizophrenia spectrum disorders. They have a 2.8–3.5 increased likelihood of being obese^2^. One of the many reasons that psychiatric patients gain weight and become overweight and obese is atypical antipsychotic drugs (AAPDs) which may affect the recovery process and quality of life^3^,^4^.

AAPDs have become the first-line medication treatment for patients with schizophrenic and schizoaffective disorders. Although AAPDs appear to be a better choice for the treatment of schizophrenic patients, they are often associated with some common and debilitating side effects, such as accelerated weight gain, insulin resistance, diabetes, hyperlipidemia, and coronary heart disease^5^,^6^,^7^. These metabolic alterations can develop as quickly as six months after the initiation of pharmacotherapy^8^. Antipsychotic-induced weight gain (AIWG) is quite commonly associated with AAPDs; more-so than typical antipsychotic drugs (TAPDs)^9^.

Although the phenomenon of antipsychotic-induced weight gain (AIWG) is well recognized, some individuals actually lose weight or their weight remains unchanged during single AAPD treatment, which shows that not all individuals are equally prone to this adverse side effect^10^. Although many variables contribute to the heterogeneity of AIWG, genetic factors may plan a significant role. Twin and sibling genetic studies have demonstrated similar degrees in weight gain profiles upon receiving AAPD^11^,^12^, and the heritability (*h*^2^) is 0.6–0.8^13^.

With the rapid development of molecular genetics, numerous pharmacogenetics studies have focused on identifying specific gene variants contributing to AIWG. Most studies have been based on the candidate gene approach, such as *HTR2C*^14^, *MC4R*^15^, *Leptin*^16^, *FTO*^17^ and *BDNF*^18^. So far, three genome-wide association studies (GWAS) of AIWG were carried out, associations were found on pro-melanin-concentrating (*PMCH*) and MC4R gene polymorphisms^19^,^20^. A recent GWAS for AIWG found that *PTPRD* polymorphisms might modulate AIWG^21^. Replications of these findings need to be carried out in independent cohorts. Compared with the GWAS design that requires very large sample sizes to achieve sufficient power, the candidate gene approach allowed us to perform an association study with increased statistical power for prioritized genes^22^.

Previous studies are mainly categorical correlation analyses. Patients with clinically significant weight gain (7% increases from baseline) were in the case group, and the others were controls. With such a research design, it is difficult to dynamically observe the quantitative traits related to AIWG. In this study, we aimed to find AIWG candidate genes in Han Chinese schizophrenia patients by studying quantitative associations between single-nucleotide polymorphisms (SNPs) of candidate genes and AIWG and other antipsychotic-treatment-related phenotypes.

## Results

### Sample demographics

Demographic characteristics of the 339 individuals, including 86 first-episode patients (FEPs), from this study are provided in Table 1. Patients received 12 weeks of treatment with single AAPDs: olanzapine (OLZ), risperidone (RIS), clozapine, quetiapine, aripiprazole, or ziprasidone.

After 12-week AAPD intervention, the average weight gain was 4.21 kg in all patients. The 12-week BMI change in our subjects (ΔBMI_12w_) was 1.52 ± 1.51 kg/m^2^, ranged from −0.78–8.36 kg/m^2^. The 12-week change in waist-to-hip ratio (ΔWHR_12w_) was 0.04 ± 0.05, ranged from −0.15–0.18. The definition of a weight gain “case” was that a patient who gained 7% or more of baseline body weight in a short-term trial^23^. The percentage of all patients meeting this criterion was 41%; 57% in OLZ group; and 24% in the RIS group. OLZ-treated patients demonstrated a markedly different distribution, with the majority of patients experiencing extreme AIWG. RIS treatment showed moderate effects on weight gain (Supplemental Figure 1).

### Quantitative association analysis

After HWE tests, SNP rs1526167 failed. In our candidate association study, the *TOX* SNP rs11777927 (*P* = 0.009) and the *ADIPOQ* rs182052 (*P* = 0.019) were significantly associated with AIWG. The *CDKN2A/B* SNPs rs3731245 (*P* = 0.04) and rs2811708 SNP (*P* = 0.039) were also associated with the AIWG; *CDKN2A/B* associations with AIWG remained significant in the FEP and RIS groups. The *BDNF* SNP rs6265 (*P* = 0.002), *BDAF* SNP rs11030104 SNP (*P* = 0.001), and *ADIPOQ* SNPs rs822396 (*P* = 0.003) and rs1501299 (*P* = 0.040) were associated with the change of WHR; consistent results were found for those SNPs in the OLZ group. These results were still significant after age adjustments (Table 2). The *BDNF* SNP rs6265 and the *BDAF* SNP rs11030104 associations remained significant after Bonferroni corrections.

Changes of lipid profiles (high- and low-density lipoprotein and total cholesterol) after AAPD treatment were linked to a *PKHD1* gene SNP rs9395706. The *CDKN2A/B* gene SNP rs10811661 was associated with the FPG changes induced by OLZ (*P* = 0.009). Results remained significant after age adjustments (Supplemental Table 1).

### Gene-gene interaction analysis

Gene × gene interaction analyses showed significant epistasis between *MTHFR* and *PCAF, EPB41L4A* and *LEPR, ADIPOQ* and *CDKN2A/B, ADIPOQ* and *NRXN3, TOX* and *PKHD1, TOX* and *RPTOR*, and *MC4R* and *COMT* gene SNPs for AIWG (ΔBMI) in all patients (Table 3).

### Genotypic association analysis

**Figure 1:**
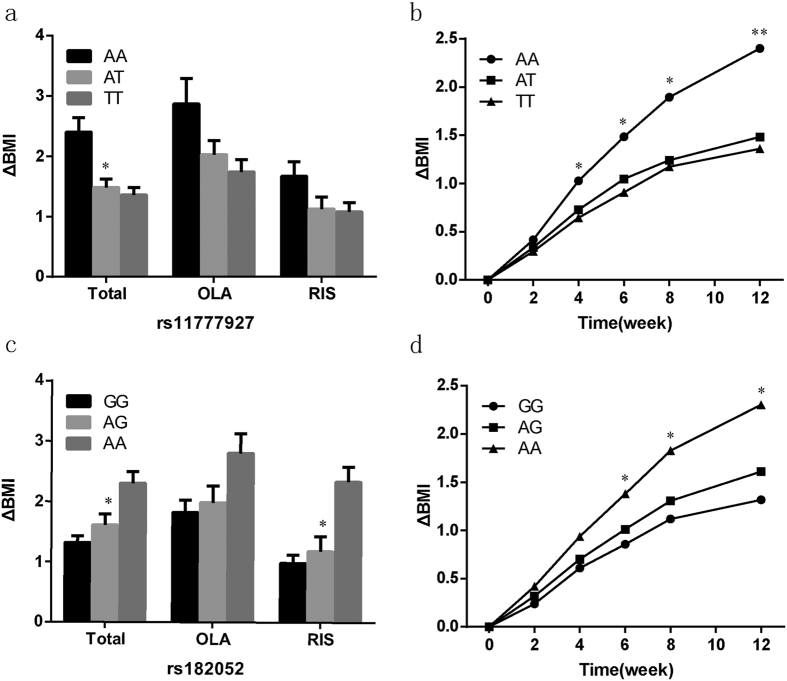
Body changes (ΔBMI) of *TOX* rs11777927 genotypes and *ADIPOQ* rs182052 genotypes. (**a**) ΔBMI at 12 weeks of *TOX* rs11777927 genotypes (AA, AT, TT) in all patients and by treatment group: olanzapine (OLZ) and risperidone (RIS). ΔBMI was compared across the three genotypes in each group (*P < 0.05, one-way ANOVA). (**b**) ΔBMI over a 12-week single AAPD treatment by *TOX* rs11777927 genotype (AA, AT, TT) in all patients. BMI was measured at baseline and at weeks 2, 4, 6, 8, and 12. ΔBMI was compared across the three genotypes at each time point (*P < 0.05, ^**^P < 0.01, one-way-ANOVA). (**c**) ΔBMI at 12 weeks of *ADIPOQ* rs182052 genotypes (GG, AG, AA) in all patients and by treatment group: OLZ and RIS. ΔBMI was compared across the three genotypes in each group (*P < 0.05, one-way-ANOVA). (**d**) ΔBMI over a 12-week single AAPD treatment by *ADIPOQ* rs182052 genotype (GG, AG, AA) in all patients. BMI was measured at baseline and at weeks 2, 4, 6, 8, and 12. ΔBMI was compared across the three genotypes at each time point (*P < 0.05, one-way-ANOVA).

Compared with patients that carried the *TOX* rs11777927 T allele, the BMI of the patients with the *TOX* rs11777927 AA genotype increased significantly after AAPD treatment (*P* < 0.05); compared with patients that carried *ADIPOQ* rs182052 genotypes GG and AG, BMI of patients with the AA genotype increased significantly (*P* < 0.05) (Fig. 1).

Compared with patients that carried *CDKN2A/B* rs3731245 genotypes CC and CT, BMI of patients with the TT genotype increased significantly (*P* < 0.05) (Supplemental Figure 2); compared with the patients that carried the *CDKN2A/B* rs2811708 G allele, the BMI of the patients with the *CDKN2A/B* rs2811708 TT genotype increased significantly after the treatment of AAPD (*P* < 0.05) (Supplemental Figure 3).

In binary association studies for BMI changes, the *TOX* gene SNP rs11777927 (*P* = 0.004), the *ADIPOQ* gene SNP rs1501299 (*P* = 0.016), the *CDKN2A/B* gene SNP rs3731245 (*P* = 0.032), rs2811708 (*P* = 0.017), the *MC4R* gene SNP rs6567160 (*P* = 0.016), and the *NRXN3* gene SNP rs12891144 (*P* = 0.023) were associated with AIWG.

## Discussion

With the development of molecular genetics during the past decade, intensive research has examined the influence of genetic variations on AIWG. However, as yet no genetic tests for AIWG are endorsed for clinical application. Although there are significant findings for AIWG associations with many other genes, the most consistently replicated findings are with *HTR2C, MC4R,* and *leptin* genes^24^,^25^. A recent meta-analysis reported that 11 SNPs from 8 genes were associated with weight or BMI change, and 4 SNPs from 2 genes were significantly related to categorical weight or BMI increase^14^. Many previous studies have not been confirmed because of varying criteria, differences in frequencies across different populations, or poor statistical power. In addition, compared with the candidate-gene-based approach, GWAS of AIWG are relatively limited^19^,^20^,^21^. Genes *PTPRD, MC4R*, and *PMCH* were discovered having the strongest associations with AIWG.

AIWG is thought to be multifactorial and polygenic, which may be different from simple obesity per se. In recent years research has pointed to a broader array of genes and pathways hypothesized to underpin AIWG. Attention has turned to genes and pathways harboring variants that might make the risk of energy unbalance increased by the influence of antipsychotics, leading to AIWG^26^. Thus, seeking AIWG genes in Han Chinese would promote the development of predictive genetic tests and help us better understand and guide treatment.

In this study, we analyzed 43 SNPs in 23 candidate genes for quantitative association with AIWG for the first time. Here, we decided to use the candidate gene approach rather than performing a GWAS to select a small set of variants for a more focused study, providing sufficient power for these selected variants given the small sample size. As candidate genes, we chose (a) genes mainly from a previous GWAS for Caucasian obesity-AIWG subjects and (b) genes associated with simple obesity, type 2 diabetes, microvascular complications of diabetes, and AIWG.

In the present study, SNPs in *TOX* and *ADIPOQ* yielded the most significant associations for AIWG, and *CDKN2A/B* showed light to strong associations. We did not replicate associations with several well-known AIWG genes, including *FTO, MTHFR*, and *COMT*, in our Han Chinese population. Potential reasons for this include genetic heterogeneity, less covered genes (i.e., too few SNPs were genotyped for certain candidate genes), entry criteria, and the relatively small sample size. Although we were unable to test genome-wide association for all genes, the spectrum of AIWG-associated genes differed between Han Chinese in our study and European populations in the previous study^15^,^16^,^17^,^27^.

The *TOX* quantitative association with AIWG was first discovered in this study. *TOX* (thymocyte selection-associated HMG box) is a member of a novel gene family and encodes a novel nuclear DNA-binding protein belonging to a large superfamily of HMG (high-mobility-group) proteins^28^,^29^. *TOX* may play a role in regulating expression of genes involved in cell cycle progression, such as the cell division cycle gene and oncogenes^30^. In recent years, emerging studies have found that *TOX* is aberrantly expressed or mutated in various diseases, such as leukemia^29^ or cardiovascular diseases^31^. A previous study by our group first found the *TOX* gene association with type 2 diabetes^32^. In the present study, we tested 3 SNPs of the *TOX* gene, rs1526167, rs2726557, and rs11777927 (Supplemental Figure 4). Unfortunately, the SNP rs1526167, which was located in a separate haplotype block, not in linkage disequilibrium with SNPs in the *TOX* gene coding region and introns, failed the HWE test.

Our results showed that the *TOX* SNP rs11777927 was significantly associated with AIWG (*P* = 0.009) and that the A allele contributed to the increased risk for AIWG. And gene × gene interaction analyses showed significant epistasis between *TOX* and *PKHD1* and between *TOX* and *RPTOR* for AIWG (ΔBMI) in all patients. We found some associations of *TOX* SNPs with obesity and metabolic-syndrome-related phenotypes. It is stated by Cox *et al*. in a published US patent application (US 2006/0177847 A1, August 10, 2006), that they found the *TOX* polymorphism and other 27 DNA sequence variations to be related to OLZ-treatment-emergent weight gain and “metabolic syndrome” in a 1.7 million SNP genome association study.

However, no rs11777927 association has been reported for AIWG. To the best of our knowledge, only one association study of rs11777927 and disease—intracranial aneurysm— has been reported^33^. The biological connections between *TOX* and AIWG are poorly understood, perhaps owing to the inflammatory responses mediated by immune cells, developmentally regulated by *TOX* gene, contributing to AIWG.

The *ADIPOQ* locus has been shown to be the only major gene for plasma adiponectin, which is exclusively expressed in adipose tissue^34^. The *ADIPOQ* SNP rs1501299 has been reported to be associated with the risk of obesity^35^,^36^ and cardiovascular diseases^37^,^38^,^39^. Four candidate SNPs in *ADIPOQ* in this study were selected from four different haplotype blocks: rs182052, rs822396, rs7649121, and rs1501299 (Supplemental Figure 5). The results showed that the *ADIPOQ* SNP rs182052 (*P* = 0.019) was significantly associated with AIWG, and that the *ADIPOQ* SNPs rs822396 (*P* = 0.003) and rs1501299 (*P* = 0.040) were associated with changes in WHR.

Other studies have not delivered consistent findings for *ADIPOQ* and AIWG. An association of rs1501299 with significant weight gain (>7% of the baseline weight) was reported in Chinese patients^40^. Another study reported genotypic or allelic association of 6 *ADIPOQ* variants with AIWG in European patients^41^. However, the latest two studies did not support a major role of *ADIPOQ* rs1501299 in the regulation of AIWG^42^,^43^, and the association of rs1501299 with AIWG was not present in Japanese patients^44^. The SNP rs1501299 was a hot spot that had attracted many researchers’ interest. Interestingly, our results showed rs1501299 was associated with changes in WHR but not in AIWG, and that rs182052 and rs822396 were significantly associated with AIWG and changes in WHR induced by AAPD, which haven’t previously been reported. *ADIPOQ* is most likely closely related to AIWG, which needs further research.

*BDNF* can encode the BDNF precursor protein located in the chromosome 11p13 region^45^. And BDNF crossing the blood-brain barrier is the most abundant neurotrophin that modulates synaptic transmission and neuroplasticity in the central nervous system^46^,^47^. Previous researches suggested that the *BDNF* rs6265 was associated with many eating disorders^48^,^49^. In addition, several SNPs in the *BDNF* and *BDAF* were strongly associated with obesity in GWAS studies^50^,^51^.

In the field of the genetics of AIWG, the recent two studies showed *BDNF* rs6265 was associated with the increased BMI in the psychiatric patients receiving AAPD^18^,^52^. Moreover, the *BDNF* haplotypes including SNP rs6265 were associated with AIWG^53^,^54^. However, a study reported that *BDNF* rs6265 was not associated with AIWG performed by Tsai *et al*.^55^. In the present study, we failed to find the association between *BDNF* rs6265 and AIWG, which was consisted with the results of Tsai’s. Interestingly, we found the *BDNF* SNP rs6265 (*P* = 0.002) and *BDAF* SNP rs11030104 SNP (*P* = 0.001) were significantly associated with the change of WHR induced by AAPD. The WHR is a alternative measure which have been found to be superior to BMI to reflect abdominal obesity in the World Health Organization (WHO) guidelines^56^.

All the above findings, including ours, indicate that the SNPs of *BDNF* have a significant impact on the obesity schizophrenic patients induced by AAPD. The associated genetic markers from *BDNF* may have different effects, and accumulated mutations may provide a whole contribution to the obesity induced by AAPD. The SNP *BDNF* rs6265 may play an important role in this process. Hence, the following study about the function of the genetic variants will be necessary to elucidate the mechanism how the genetic variants in *BDNF* medicate signaling pathway and lead to obesity.

*CDKN2A/B* is located in the chromosome 9p21 region, has been highlighted as the strongest genetic susceptibility locus for cardiovascular disease^57^,^58^ and type 2 diabetes^58^,^59^. *CDKN2A/B* encodes the CDK inhibitor proteins involved in cell cycle regulation, aging, senescence, and apoptosis. In the present study, we selected 3 SNPs of *CDKN2A/B*: rs3731245, rs2811708 and rs10811661 (Supplemental Figure 6); rs3731245 and rs2811708 are in a haplotype block. SNPs rs3731245 (*P* = 0.04) and rs2811708 SNP (*P* = 0.039) were associated with the AIWG but with low statistical power. We found no association between rs10811661 and AIWG, but this SNP was associated with the FPG changes that induced by OLZ (*P* = 0.009). These results suggest that glucose and lipid metabolism abnormalities and weight gain are influenced by AAPD through different pathways. Previous studies have found that AAPD may not affect glucose and lipid metabolism directly through weight gain^10^,^60^. The mechanism by which the *CDKN2A/B* gene affects susceptibility for AIWG remains to be investigated.

In the future, we will further substantiate our gene results and explore more genetic factors underlying AIWG. However, the heterogeneity of medications and psychiatric disorder factors still exist. We analyzed the genetic association in FEPs, OLZ-treated patients, and RIS-treated patients, but the subgroups were too small for strong statistical power. For this reason, a better designed trial with different AAPDs in larger samples and different populations should also be implemented to validate the genes associated with AIWG.

Given the sample size of our study, we have moderate power to detect major AIWG associations. We performed power calculations by GPC (Genetic Power Calculator^61^), given a total QTL (quantitative trait locus) variance = 0.05, QTL increaser allele frequency = 0.25, marker allele frequency = 0.25, dominant model, total LD (linkage disequilibrium, D’ = 0.95), case trait threshold (0–4 standard deviations), control threshold (0 to −1.5), type I error α = 0.05, we have more than 80% (84%) power.

We also performed binary association studies for AIWG, the discrete analyses showed similar associations with quantitative BMI changes. Replication is essential for association studies, although GWASs for AIWG were relatively limited. We compared our associations with published GWAS for body weight related traits (GWASdb, v2. http://jjwanglab.org/gwasdb), ADIPOQ (rs182052), BDNF (rs6265), and BDAF (rs11030104) gene SNPs all yielded genome-wide associations.

In our study, we used quantitative associations between SNPs of candidate genes and AIWG and other antipsychotic-related phenotypes to find AIWG candidate genes in Han Chinese schizophrenia patients. We measured not only weight but also the WHR and lipid and glycemic profiles, which are a common index of metabolic syndrome. In addition, for the purpose of excluding environmental factors that may affected AIWG^62^, we selected a sample of inpatients with lifestyle, exercise, and diet under unified management by the hospital in order to reduce the heterogeneity. Our comprehensive study of *TOX* and *ADIPOQ* is an important contribution to understanding the biology of AIWG.

## Patients and Materials

### Study participants and design

A total of 339 patients hospitalized with schizophrenia or schizoaffective disorders were recruited. All the subjects were unrelated Han Chinese collected from the Tianjin Metal Health Centre, which is Asia’s largest independent psychiatric hospital. The inclusion criteria for this study were as follows: (a) Clinical diagnoses were independently confirmed by two psychiatrists according to the *Diagnostic and Statistical Manual of Mental Disorders*, fourth edition, text revision. (b) All patients were first-episode drug-naive patients or without any antipsychotic drugs at least 4 weeks before enrollment. (c) The patients were physically healthy with normal hematological and biochemical parameters. (d) All patients were 18–60 years old. Subjects with neurological disorders, eating disorders, and thyroid diseases were excluded from the study. Clinical characteristics of patients were shown in Table 1. All subjects provided written informed consent prior for this study, and the protocol was approved by the Committee on Studies Involving Human Beings at Tianjin Medical University. All experiments were performed in accordance with relevant guidelines and regulations.

All patients received single AAPD intervention on the basis of clinical treatment need. Only trihexyphenidyl for extrapyramidal symptoms and lorazepam for insomnia or agitation were allowed as needed during the study period as concomitant medications.

Patients were followed up during the 12-week treatment course. All patients were measured for body weight and for waist, abdominal, and hip circumferences at baseline and at weeks 2, 4, 6, 8 and 12 after treatment initiation. Body mass index (BMI) and waist-to-hip ratio (WHR) were calculated. Before and after treatment for 4, 8, and 12 weeks, triglyceride, high- and low-density lipoprotein, total cholesterol, total protein, albumin, fasting plasma glucose (FPG), blood creatinine, urea nitrogen (urea), urea/creatinine ratio, and serum C reactive protein were measured for all patients.

### Candidate gene and SNP selection

Candidate genes mainly came from a comparative GWAS for Caucasian obesity-AIWG subjects performed by our project team members and colleagues. Genes associated with simple obesity, type 2 diabetes, or microvascular complications of diabetes in our previous studies were also included^32^,^63^, in a GWAS of simple obesity, which was the largest sample size by far (340,000 persons)^51^, and in other previous candidate gene associations for AIWG that gave inconsistent or controversial results^15^,^16^,^17^,^18^,^27^. Forty-three (43) SNPs in 23 candidate genes were selected in our study (Table 4).

For some genes, tagging SNPs were selected using the HapMap database (phase2 + phase3, release #28, CEU population, Build36; www.hapmap.org) and Tagger in Haploview^64^. Minor allele frequencies of Han Chinese were taken from dbSNP (http://www.ncbi.nlm.nih.gov/snp/). For previously reported associations, we selected SNPs with the most significant association rather than genotyping the whole gene. For less studied genes, multiple SNPs were chosen based on the LD pattern of the gene (r^2^ > 0.8).

### Genotyping and quality control

Genomic DNA samples were extracted from 5 ml of peripheral whole blood samples using the high-salt method. Samples were stored and processed by the Center for Molecular and Population Genetics at Tianjin Medical University. Genotyping was performed by primer extension of multiplex products with detection by matrix-assisted laser desorption time-of-flight mass spectrometry. For quality control, 10% of the sample was randomly re-genotyped, with a 100% concordant rate. All genotyping was done blind to knowledge of subjects’ clinical data.

### Statistical analysis

The Hardy-Weinberg equilibrium (HWE) test was carried out before the association analysis (Table 4). All phenotypes were documented in a Filemaker Pro database. Statistical analyses for phenotypes were performed by SPSS software (version 20.0). We tested for recessive effects for three significant SNPs by comparing the three genotypes or different subgroups using ANOVA, with change in BMI across the 12-week trial as the dependent measure. The level of statistical significance for the above tests was set a priori at *P* < 0.05.

The linear regression model in the PLINK^65^ was used to test the association between the chosen SNPs and several phenotypes, where the changes of weight, BMI, WHR, lipid and glycemic profiles were used as phenotypes for the quantitative trait locus analyses. Linear regressions were performed for each quantitative trait against age within sexes, standardized residuals were saved to make mean = 0 and standard deviation = 1 for each phenotype. Outliers (more than 4 standard deviations) were deleted from this study. As 43 SNPs in 23 candidate genes were analyzed, we used Bonferroni corrections for multiple testing, and the nominal *P*-value should be at least 0.002 to be significant after multiple test corrections.

Pairwise gene-gene interaction analyses (epistasis) were carried out by PLINK^65^ among candidate gene SNPs in all patients, the FEP group, the OLZ treatment group, and the RIS treatment group, Bonferroni corrections were also employed for multiple testing.

In addition, we also performed a discrete association study for AIWG by PLINK. A weight gain “case” was defined as a patient who gained 7% or more of his or her baseline body weight in a 12-week trial.

## Conclusions

In Conclusion, our findings suggest the role of *TOX, ADIPOQ* and *BDNF* in weight and WHR gain induced by atypical antipsychotics in schizophrenia subjects. This study provides a better understanding of genetic factors predisposing individuals to AAPD-induced obesity, dyslipidemia, and abnormal glucose metabolism that may help guide clinical medication intervention and also reveal the pathogenesis of AIWG.

## Additional Information

**How to cite this article:** Li, S. *et al. TOX* and *ADIPOQ* Gene Polymorphisms Are Associated with Antipsychotic-Induced Weight Gain in Han Chinese. *Sci. Rep.* **7**, 45203; doi: 10.1038/srep45203 (2017).

**Publisher's note:** Springer Nature remains neutral with regard to jurisdictional claims in published maps and institutional affiliations.

## Supplementary Material

Supplementary Materials

## Figures and Tables

**Table 1:** Clinical characteristics of subjects.

Characteristic	N (%)/Mean ± SD
Gender	
Male	135 (39.8)
Female	204 (60.2)
Age (years)	38.7 ± 11.53
Duration of illness (years)	9.31 ± 8.67
First-episode patients (FEPs)	86 (25.4)
BMI at baseline	23.42 ± 4.10
WHR at baseline	0.88 ± 0.06
Medication	
Olanzapine	131 (38.6)
Risperidone	133 (39.2)
Clozapine	23 (6.8)
Quetiapine	27 (8.0)
Aripiprazole	14 (4.1)
Ziprasidone	11 (3.2)

Abbreviations: BMI, body mass index; WHR, Waist -Hip Ratio.

**Table 2:** Quantitative association analysis of candidate genes and changes in phenotype at 12 weeks.

SNP	Gene	Risk allele	*P*	*P* (adjusted^a^)
**All patients**				
ΔBMI				
rs182052	*ADIPOQ*	A	0.019	0.019
rs11777927	*TOX*	A	0.009	0.009
rs351320	*EPHA7*	G	0.025	0.035
rs3731245	*CDKN2A/B*	A	0.040	0.040
rs2811708	*CDKN2A/B*	T	0.039	0.044
ΔWHR				
rs822396	*ADIPOQ*	G	0.003	0.005
rs1501299	*ADIPOQ*	A	0.040	0.045
rs6265	*BDNF*	G	0.002	0.002
rs11030104	*BDAF*	G	0.001	0.002
**FEP group**^b^				
ΔBMI				
rs279858	*GABRA2*	G	0.029	—
rs249496	*PCAF*	A	0.019	—
rs2811708	*CDKN2A/B*	T	0.028	—
rs6567160	*MC4R*	C	0.041	—
ΔWHR				
rs35747495	*PCAF*	T	0.024	—
rs10811661	*CDKN2A/B*	C	0.031	—
rs489693	*MC4R*	A	0.011	—
**OLZ group**^b^				
ΔBMI				
rs279858	*GABRA2*	G	0.021	—
rs10968576	*LINGO2*	G	0.020	—
ΔWHR				
rs822396	*ADIPOQ*	G	0.047	—
rs6265	*BDNF*	G	0.020	—
rs11030104	*BDAF*	G	0.020	—
**RIS group**^b^				
ΔBMI				
rs3776871	*PAM*	A	0.039	—
rs7799039	*LEP*	G	0.002	—
rs3731245	*CDKN2A/B*	A	0.006	—
rs2811708	*CDKN2A/B*	T	0.002	—
rs6567160	*MC4R*	C	0.006	—
rs489693	*MC4R*	A	0.018	—

ΔBMI, change in body mass index; ΔWHR, change in waist-to-hip ratio.

^a^All the dependent variables were adjusted for age and gender.

^b^No age and gender adjusted phenotypes were used in subgroups due to limited sample size.

**Table 3:** Quantitative gene-gene interaction analysis of AIWG (ΔBMI).

Gene 1	SNP1	Gene 2	SNP2	*P*	*P* (adjusted^a^)
**All patients**					
* MTHFR*	rs1801131	*PCAF*	rs1986917	4.79 × 10^−3^	5.71 × 10^−4^
* LEPR*	rs1137101	*EPB41L4A*	rs7732687	2.08 × 10^−3^	2.33 × 10^−3^
* ADIPOQ*	rs182052	*NRXN3*	rs11845632	7.29 × 10^−3^	7.53 × 10^−3^
* ADIPOQ*	rs822396	*CDKN2A/B*	rs3731245	8.29 × 10^−3^	3.09 × 10^−3^
* ADIPOQ*	rs7649121	*CDKN2A/B*	rs3731245	6.91 × 10^−4^	6.37 × 10^−4^
* ADIPOQ*	rs7649121	*CDKN2A/B*	rs2811708	4.19 × 10^−4^	3.46 × 10^−4^
* GABRA2*	rs279858	*RPTOR*	rs12940622	2.84 × 10^−3^	5.18 × 10^−3^
* PKHD1*	rs2894788	*TOX*	rs2726557	6.21 × 10^−3^	5.64 × 10^−3^
* TOX*	rs2726557	*RPTOR*	rs12940622	9.21 × 10^−3^	7.52 × 10^−3^
* PTPRD*	rs10977144	*CDKN2A/B*	rs10811661	8.31 × 10^−3^	8.33 × 10^−3^
* MC4R*	rs489693	*COMT*	rs4680	5.63 × 10^−3^	6.20 × 10^−3^
**FEP group**^b^					
* MTHFR*	rs1801131	*EPB41L4A*	rs7732687	7.98 × 10^−3^	—
* LEPR*	rs1137101	*PCAF*	rs1986917	3.74 × 10^−3^	—
* LEPR*	rs1137101	*EPB41L4A*	rs7732687	8.15 × 10^−3^	—
* INSIG2*	rs7566605	*NRXN3*	rs11845632	1.23 × 10^−3^	—
* PCAF*	rs1986917	*ADIPOQ*	rs7649121	7.13 × 10^−4^	—
* PCAF*	rs1986917	*LINGO2*	rs10968576	5.24 × 10^−3^	—
* ADIPOQ*	rs7649121	*PKHD1*	rs9296661	7.23 × 10^−3^	—
* ADIPOQ*	rs7649121	*PKHD1*	rs1326589	7.23 × 10^−3^	—
* ADIPOQ*	rs7649121	*NRXN3*	rs12891144	3.88 × 10^−4^	—
* PAM*	rs249496	*NRXN3*	rs12891144	6.37 × 10^−3^	—
* PKHD1*	rs2894788	*PAPPA*	rs10817871	6.54 × 10^−3^	—
* CNR1*	rs806368	*EPHA7*	rs351320	2.70 × 10^−3^	—
* BDNF*	rs6265	*NRXN3*	rs11845632	1.75 × 10^−3^	—
* BDAF*	rs11030104	*NRXN3*	rs11845632	3.13 × 10^−3^	—
**OLZ group**^b^					
* INSIG2*	rs7566605	*TOX*	rs11777927	9.38 × 10^−3^	—

^a^All the dependent variables were adjusted for age and gender.

^b^No age and gender adjusted phenotypes were used in subgroups due to limited sample size.

**Table 4:** Candidate genes and SNPs genotyped in this study.

Chr	SNP	Base-Pair Position	Gene	HWE (*P*)	MAF CHB*	Location
1	rs1801133	11856378	*MTHFR*	0.165	0.49 (C)	Intragenic
1	rs1801131	11854476	*MTHFR*	0.390	0.20 (C)	Intragenic
1	rs1137101	66058513	*LEPR*	0.171	0.11 (A)	Intron
2	rs7566605	118836025	*INSIG2*	0.287	0.36 (G)	Intergenic
3	rs2929402	20096110	*PCAF*	0.348	0.49 (A)	Intergenic
3	rs35747495	20077262	*PCAF*	0.273	0.32 (T)	Intron
3	rs1986917	20118522	*PCAF*	0.723	0.43 (C)	Intron
3	rs1501299	186571123	*ADIPOQ*	0.053	0.33 (A)	Intron
3	rs182052	186560782	*ADIPOQ*	0.338	0.47 (A)	Intron
3	rs822396	186566877	*ADIPOQ*	0.407	0.11 (G)	Intron
3	rs7649121	186568785	*ADIPOQ*	0.216	0.24 (T)	Intron
4	rs279858	46314593	*GABRA2*	0.120	0.48 (G)	Intragenic
5	rs3776871	102292211	*PAM*	0.689	0.39 (A)	Intron
5	rs249496	102201590	*PAM*	0.725	0.29 (G)	UTR 5
5	rs7732687	111571642	*EPB41L4A*	0.643	0.47 (C)	Intron
6	rs351320	94019904	*EPHA7*	0.057	0.4 (G)	Intron
6	rs164547	94071318	*EPHA7*	0.113	0.44 (T)	Intron
6	rs1326589	51674161	*PKHD1*	0.052	0.25 (T)	Intron
6	rs2894788	51643268	*PKHD1*	0.191	0.22 (C)	Intron
6	rs9296661	51673439	*PKHD1*	0.052	0.23 (G)	Intron
6	rs9395706	51544360	*PKHD1*	0.477	0.41 (G)	Intron
6	rs806368	88850100	*CNR1*	0.105	0.49 (C)	UTR 3
7	rs7799039	127878783	*LEP*	0.057	0.23 (G)	Intergenic
8	rs1526167	59702355	*TOX*	0.000	0.48 (G)	Intergenic
8	rs2726557	59792800	*TOX*	0.808	0.49 (G)	Intron
8	rs11777927	59881039	*TOX*	0.286	0.38 (A)	Intron
9	rs10968576	28414339	*LINGO2*	0.557	0.22 (G)	Intron
9	rs10811661	22134094	*CDKN2A/B*	0.466	0.43 (C)	Intergenic
9	rs2811708	21973422	*CDKN2A/B*	0.246	0.22 (T)	Intron
9	rs3731245	21972445	*CDKN2A/B*	0.072	0.20 (A)	Intron
9	rs10977144	8474233	*PTPRD*	0.741	0.11 (T)	Intron
11	rs11030104	27684517	*BDAF*	0.370	0.41 (G)	Intron
11	rs6265	27679916	*BDNF*	0.400	0.37 (G)	Unknown
14	rs12891144	79945552	*NRXN3*	0.458	0.17 (C)	Intron
14	rs11845632	79953814	*NRXN3*	0.827	0.34 (A)	Intron
14	rs7141420	79899454	*NRXN3*	0.900	0.36 (T)	Intron
16	rs9939609	53820527	*FTO*	0.072	0.12 (T)	Intron
16	rs1558902	53803574	*FTO*	0.058	0.12 (A)	Intron
17	rs12940622	78615571	*RPTOR*	0.330	0.34 (A)	Intron
18	rs6567160	57829135	*MC4R*	0.821	0.14 (C)	Intergenic
18	rs489693	57882787	*MC4R*	0.100	0.19 (A)	Intergenic
19	rs10423928	46182304	*GIPR*	0.471	0.21 (A)	Intron
22	rs4680	19951271	*COMT*	0.773	0.31 (A)	Intragenic

*MAF, minor allele frequencies, taken from dbSNP; CHB, Han Chinese.
