# Supplementary material for: TOX and ADIPOQ Gene Polymorphisms Are Associated with Antipsychotic-Induced Weight Gain in Han Chinese
Source: Sci Rep. 2017 Mar 22;7:45203. doi: 10.1038/srep45203 (PMC5361121; doi:10.1038/srep45203)
Supplement: Supplementary Materials [file srep45203-s1.doc]

***Supplementary Materials***

***TOX* and *ADIPOQ* Gene Polymorphisms Are Associated with** **Antipsychotic-Induced Weight Gain in Han Chinese**

Shen Li^1,3^, Chengai Xu^1,2^, Yuan Tian^1^, Xueshi Wang^1,2^, Rui Jiang^2^, Miaomiao Zhang^1^, Lili Wang^2^, Guifu Yang^4^, Ying Gao^2^, Chenyu Song^1^, Yukun He^1^, Ying Zhang^2^, Jie Li^2#^, Wei-Dong Li^1#^


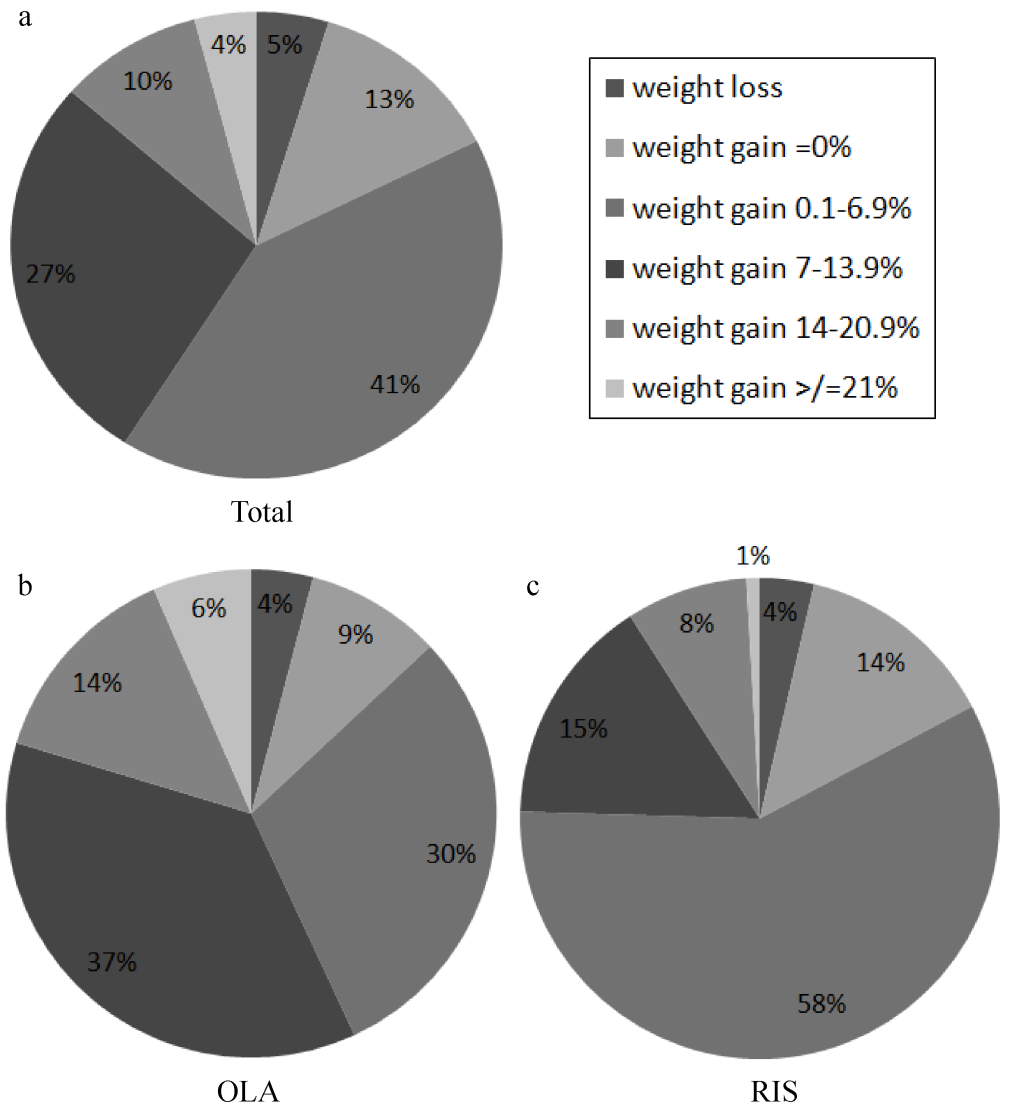


**Supplemental Figure 1** Distribution of antipsychotic-drug-induced weight gain in schizophrenic patients following a 12-week treatment with atypical antipsychotic drugs: all patients (a), the olanzapine (OLZ) group (b), and the risperidone (RIS) group (c).

**Supplemental Table 1** **Quantitative association analysis of candidate genes and the changes of lipid and glycemic profiles at 8 weeks**

| Group/Phenotype | SNP | Gene | Risk allele | *P* | *P*（adjusted^a^） |
| --- | --- | --- | --- | --- | --- |
| **Total** |  |  |  |  |  |
| ΔLDL | rs9395706 | *PKHD1* | G | 0.003 | 0.003 |
| ΔHDL | rs1801131 | *MTHFR* | C | 0.049 | 0.068 |
|  | rs279858 | *GABRA2* | G | 0.021 | 0.021 |
|  | rs3776871 | *PAM* | A | 0.047 | 0.049 |
|  | rs9395706 | *PKHD1* | G | 0.035 | 0.031 |
|  | rs12940622 | *RPTOR* | A | 0.040 | 0.033 |
| ΔCHOL | rs1801133 | *MTHFR* | C | 0.039 | 0.038 |
|  | rs351320 | *EPHA7* | G | 0.046 | 0.059 |
| ΔTG | rs351320 | *EPHA7* | G | 0.031 | 0.206 |
|  | rs12891144 | *NRXN3* | C | 0.033 | 0.072 |
| **FEP Group** |  |  |  |  | b |
| ΔLDL | rs1137101 | *LEPR* | A | 0.037 | - |
|  | rs9939609 | *FTO* | T | 0.012 | - |
|  | rs9395706 | *PKHD1* | G | 0.013 | - |
| ΔHDL | rs9395706 | *PKHD1* | G | 0.033 | - |
| ΔCHOL | rs7799039 | *LEP* | G | 0.019 | - |
| ΔTG | rs7799039 | *LEP* | G | 0.044 | - |
|  | rs10811661 | *CDKN2A/B* | C | 0.025 | - |
|  | rs6567160 | *MC4R* | C | 0.019 | - |
|  | rs489693 | *MC4R* | A | 0.016 | - |
| ΔFPG | rs1801131 | *MTHFR* | C | 0.037 | - |
|  | rs1137101 | *LEPR* | A | 0.007 | - |
|  | rs806368 | *CNR1* | C | 0.021 | - |
| **OLA Group** |  |  |  |  | b |
| ΔLDL | rs1801133 | *MTHFR* | C | 0.010 | - |
|  | rs1137101 | *LEPR* | A | 0.034 | - |
|  | rs9395706 | *PKHD1* | G | 0.033 | - |
| ΔHDL | rs279858 | *GABRA2* | G | 0.026 | - |
|  | rs3776871 | *PAM* | A | 0.011 | - |
|  | rs9395706 | *PKHD1* | G | 0.001 | - |
| ΔCHOL | rs1801133 | *MTHFR* | C | 0.006 | - |
|  | rs3776871 | *PAM* | A | 0.029 | - |
|  | rs9395706 | *PKHD1* | G | 0.035 | - |
| ΔTG | rs1801133 | *MTHFR* | C | 0.018 | - |
| ΔFPG | rs1137101 | *LEPR* | A | 0.040 | - |
|  | rs182052 | *ADIPOQ* | A | 0.045 | - |
|  | rs10811661 | *CDKN2A/B* | C | 0.009 | - |
|  | rs12940622 | *RPTOR* | A | 0.018 | - |
| **RIS Group** |  |  |  |  | b |
| ΔHDL | rs164547 | *EPHA7* | T | 0.008 | - |
|  | rs7799039 | *LEP* | G | 0.005 | - |
|  | rs4680 | *COMT* | A | 0.016 | - |
| ΔCHOL | rs3731245 | *CDKN2A/B* | A | 0.006 | - |
|  | rs7799039 | *LEP* | G | 0.028 | - |
| ΔTG | rs4680 | *COMT* | A | 0.037 | - |
| ΔFPG | rs7649121 | *ADIPOQ* | T | 0.033 | - |

CHOL, cholesterol; FPG, fasting plasma glucose; HDL, high-density lipoprotein; LDL, low-density lipoprotein; TG, triglycerides.

^a^ All the dependent variables were controlled for age and gender as confounding factors.

^b^ Because the sample size was reduced after the grouping, the residual error adjustment was not carried out.


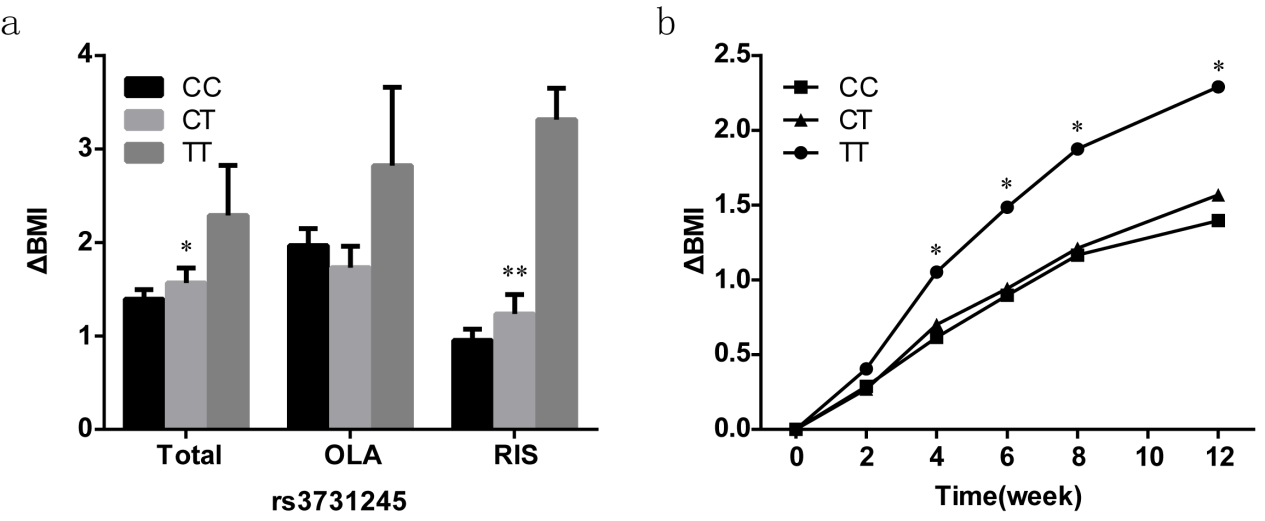


**Supplemental Figure 2** ΔBMI for *CDKN2A/B* rs3731245 genotypes. (a) ΔBMI at 12 weeks by *CDKN2A/B* rs3731245 genotype (CC, CT, TT) in all patients, the OLA group, and the RIS group. ΔBMI was compared among the three genotypes in each group (**P*<0.05, ^**^*P*<0.01, one-way ANOVA). (b) ΔBMI over the course of 12 weeks of single AAPD treatment by *CDKN2A/B* rs3731245 genotype (CC, CT, TT) in all patients. BMI was measured at baseline and at weeks 2, 4, 6, 8, and 12. ΔBMI was compared among the three genotypes at each time point (**P*<0.05, one-way ANOVA).


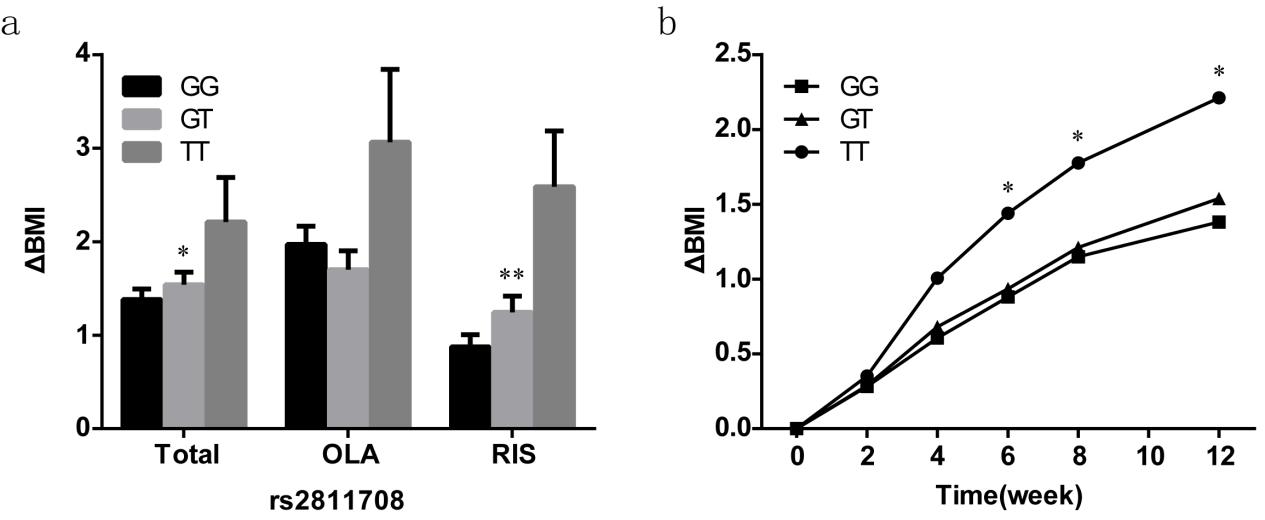


**Supplemental Figure 3** ΔBMI of *CDKN2A/B* rs2811708 genotypes. (a) ΔBMI at 12 weeks by *CDKN2A/B* rs2811708 genotype (GG, GT, TT) in all patients and in the OLA and RIS groups. ΔBMI was compared among the three genotypes in each group (**P*<0.05, ^**^*P*<0.01, one-way ANOVA). (b) ΔBMI over the course of 12 weeks of single AAPD treatment by *CDKN2A/B*rs2811708 genotype (GG, GT, TT) in all patients. BMI was measured at baseline and at weeks 2, 4, 6, 8, and 12. ΔBMI was compared among the three genotypes at each time point (**P*<0.05, one-way ANOVA).


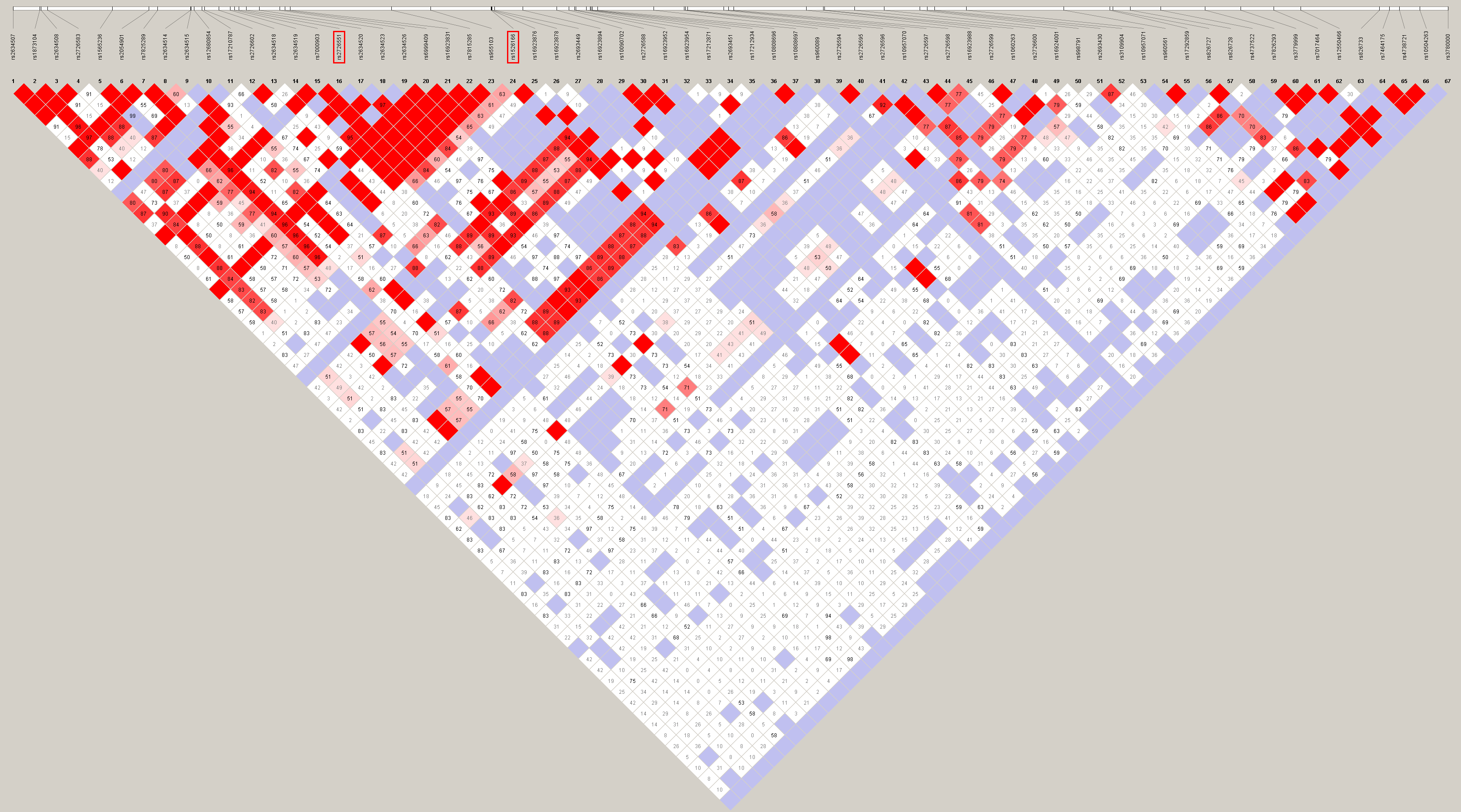


**Supplemental Figure 4** Linkage disequilibrium (r^2^ values) and haplotype blocks of *TOX* gene region SNPs (Chinese, CHB).


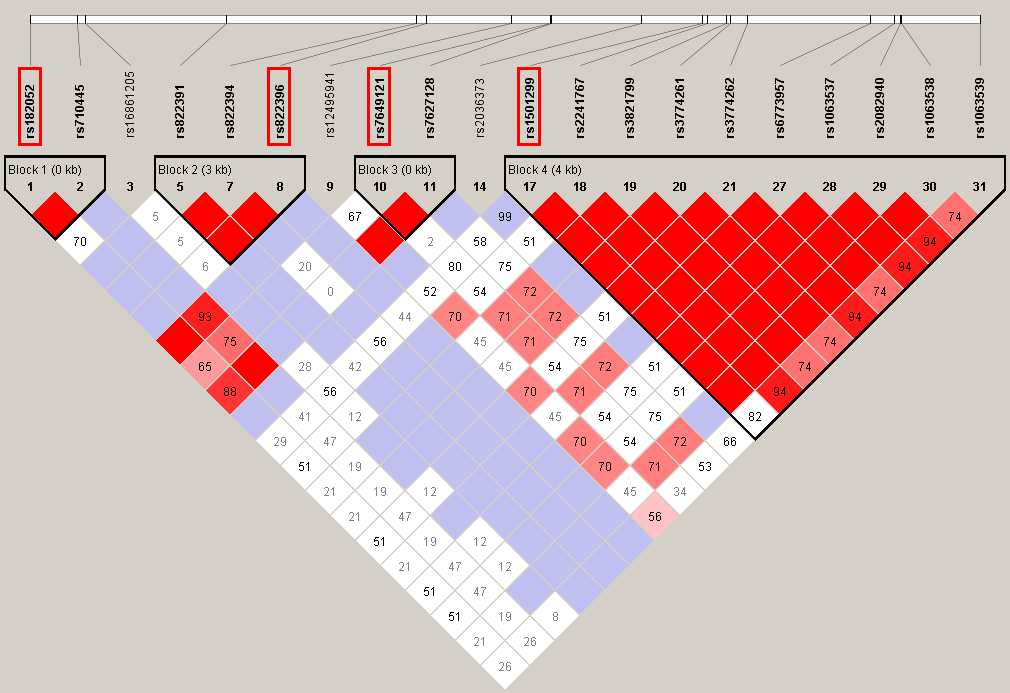


**Supplemental Figure 5** Linkage disequilibrium (r^2^ values) and haplotype blocks of *ADIPOQ* gene region SNPs (Chinese, CHB).


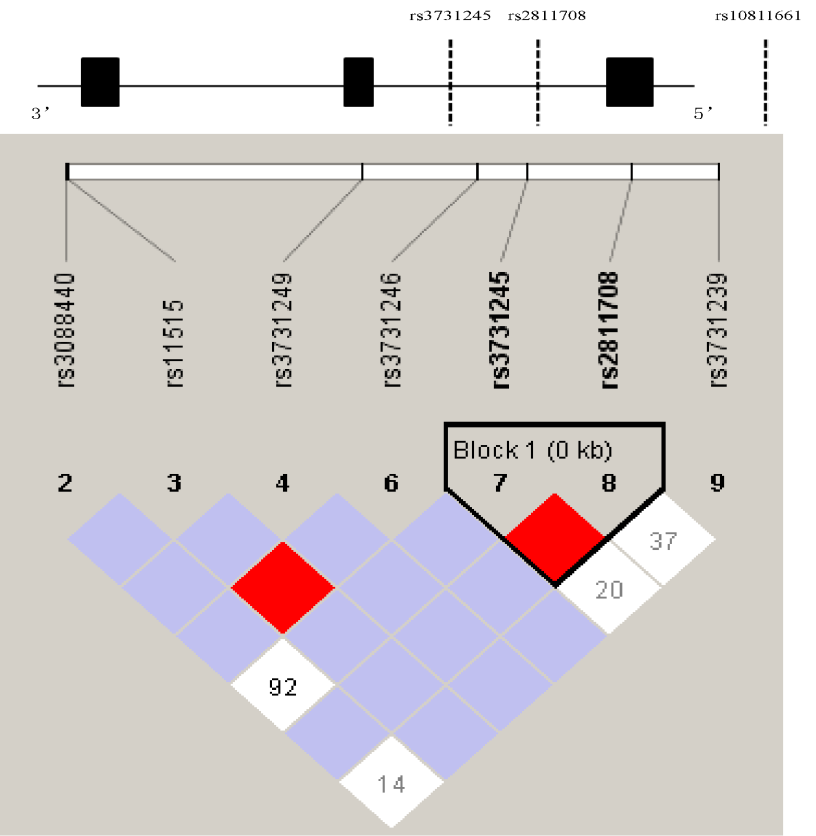


**Supplemental Figure 6** Linkage disequilibrium (r^2^ values) and haplotype blocks of the *CDKN2A/B* gene region SNPs (Chinese, CHB).
